# Breaking the Paradigm: Successful Treatment of Rare CD4/CD8 Dual-Positive, Advanced Mycosis Fungoides Using CHOP Therapy

**DOI:** 10.7759/cureus.90159

**Published:** 2025-08-15

**Authors:** Cecilia Arias Sepúlveda, Diego E Gómez López, Kenia Hernandez Rivera, Circe Ancona Castro

**Affiliations:** 1 Internal Medicine Residency, Autonomous University of Chihuahua/Institute for Social Security and Services for State Workers (ISSSTE) President Lazaro Cárdenas, Chihuahua, MEX; 2 Dermatology, Institute for Social Security and Services for State Workers (ISSSTE) Monterrey Regional Hospital, Monterrey, MEX

**Keywords:** advanced-stage mf, cd4/cd8 dual-positive, chop therapy, cutaneous t-cell lymphoma (ctcl), mycosis fungoides (mf)

## Abstract

Mycosis fungoides (MF) is one of the most important representatives of cutaneous T-cell lymphomas. This disease is rare, and the diagnosis always requires a clinical-pathological correlation. The prognosis depends on its stage and is usually favorable when found in early stages. CD4/CD8 duality is an uncommon immune phenotype. We are presenting a successful treatment of a case of dual-positive MF in the third stage. The simultaneous expression of CD4 and CD8 in skin lesions has been suggested to potentially indicate an improved clinical outcome in patients with MF. Patient was treated with the CHOP regimen and a combination of symptomatic therapies, which resulted in a favorable therapeutic response.

## Introduction

Mycosis fungoides (MF) is a rare, chronic form of skin lymphoma that originates from T-lymphocytes, a type of white blood cell involved in the immune response. It usually begins with patches or plaques on the skin that can easily be mistaken for benign conditions such as eczema or psoriasis, which often delays diagnosis. Over time, the disease may progress to tumor formation or spread to lymph nodes and internal organs. Early detection is crucial, as prognosis is generally favorable in the initial stages but worsens with advanced disease.

Cutaneous T-cell lymphomas (CTCLs) represent a subset of non-Hodgkin lymphomas that predominantly involve the skin, but may occasionally spread to the bloodstream and lymphatic tissues. The most well-known subtypes include MF and Sézary syndrome (SS). Staging of MF follows the TNMB classification, with the assessment of cutaneous (T), nodal (N), visceral (M), and hematologic (B) compartments [1]. Cutaneous extent and evidence of systemic dissemination are among the strongest predictors of prognosis. [2]

Histologically, MF is characterized by dermal infiltration of memory-type helper T cells, which are typically CD3+, CD4+, CD45RO+, and CD8−, as demonstrated through immunohistochemical analysis [3]. CD4 and CD8 are surface glycoproteins that define functionally distinct subsets of T-lymphocytes: CD4+ cells act as helper T cells, while CD8+ cells are cytotoxic. In MF, co-expression of CD4 and CD8 in the same malignant T-cell population is unusual and reported in a small minority of cases.

A review of the literature indicates that CD4/CD8 dual-positive MF is rare, with most series reporting only isolated cases or prevalence below 5% of MF diagnoses [3,4]. Its prognostic significance remains debated: some studies suggest slower progression and better outcomes, while others find no significant difference [3,5].

## Case presentation

A 55-year-old male patient with chronic comorbidities, specifically chronic kidney disease (CKD) and type 2 diabetes mellitus (DM2), presented initially with a progressive dermatosis (non-described by the patient), localized to his left thigh, first noticed in 2019. The lesion began as an erythematous macule, evolved into an infiltrated plaque, and later became an ulcerated nodular lesion that did not respond to topical steroids. At the time of initial presentation, the patient denied systemic “B” symptoms such as fever, night sweats, or unintentional weight loss. Baseline laboratory evaluation, including complete blood count, was within normal limits; lactate dehydrogenase (LDH) levels were not elevated; and HIV testing was negative.

The differential diagnosis considered included chronic plaque psoriasis, nummular eczema, and other cutaneous T-cell lymphoma variants, which were subsequently excluded based on histopathology and immunohistochemistry. Subsequently, the patient was lost to follow-up for approximately three years.

**Figure 1:**
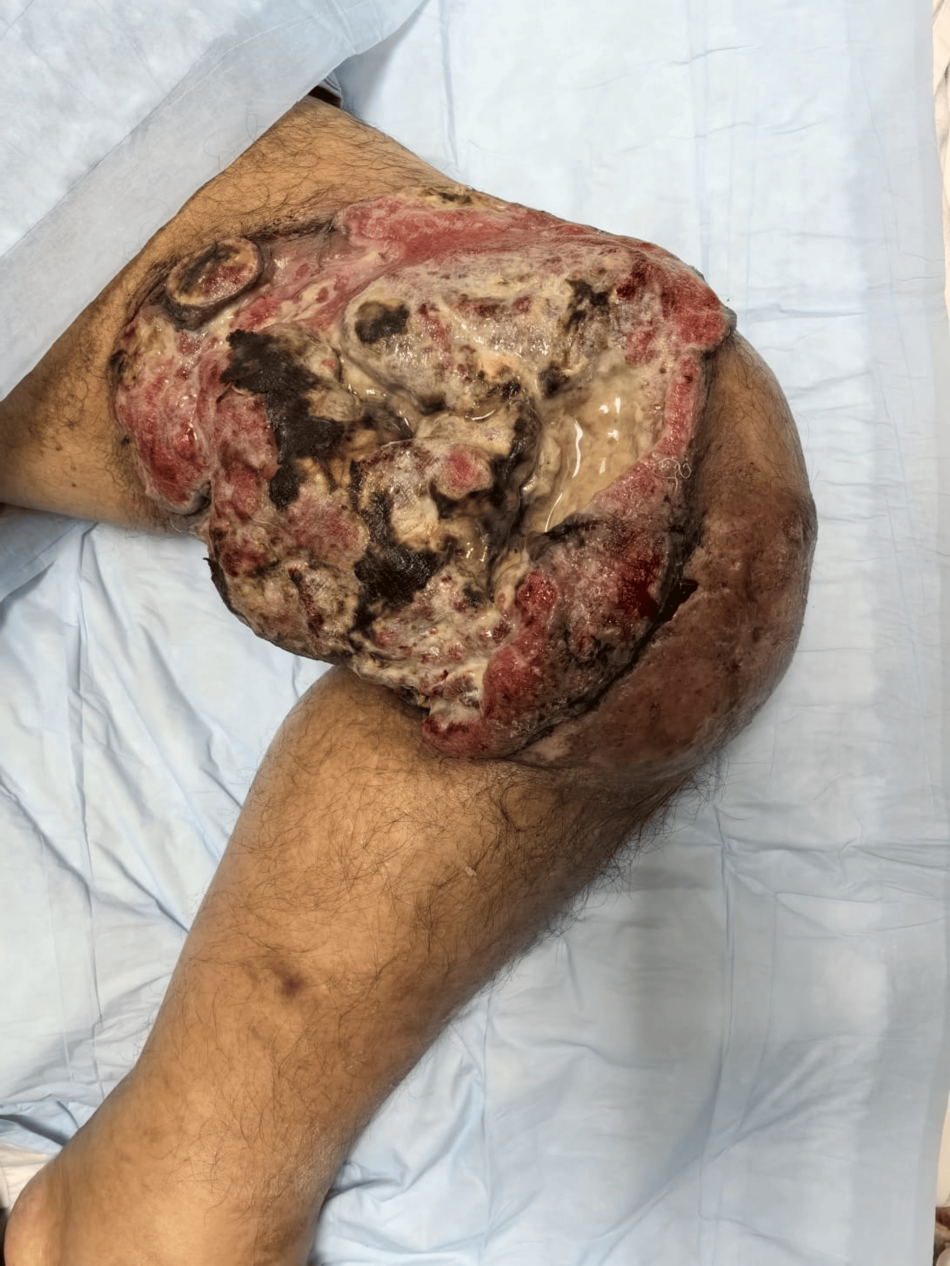
Dermatosis at admission. Dermatosis localized on the left thigh, characterized as an infected, ulcerated nodular lesion measuring approximately 12 × 18 cm.

**Figure 2:**
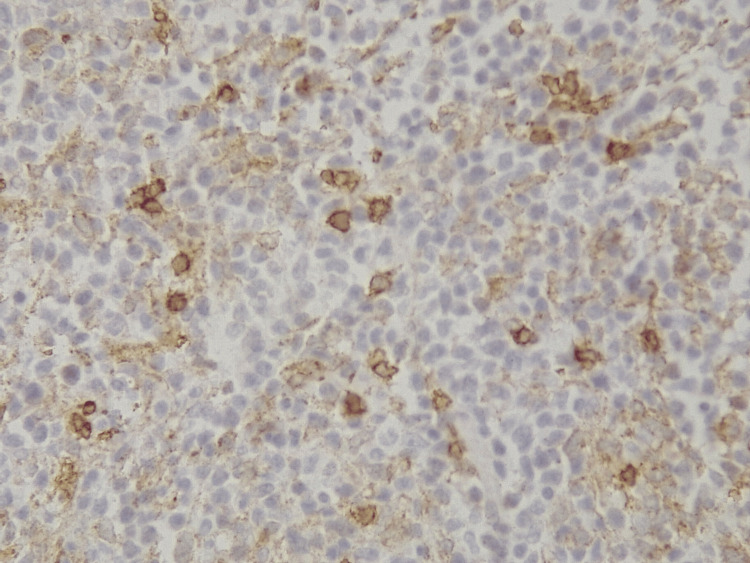
Immunohistochemistry – CD4 Immunohistochemistry for CD4 showing strong membranous staining in abnormal lymphocytes (brown chromogen) within the dermis, with hematoxylin counterstain. Approximately 20% of the lymphoid population is positive, with an intensity of 80%. CD4 positivity supports a helper T-cell phenotype in the neoplastic infiltrate. Skin biopsy from the ulcerated nodular lesion on the left thigh. Original magnification ×400.

**Figure 3:**
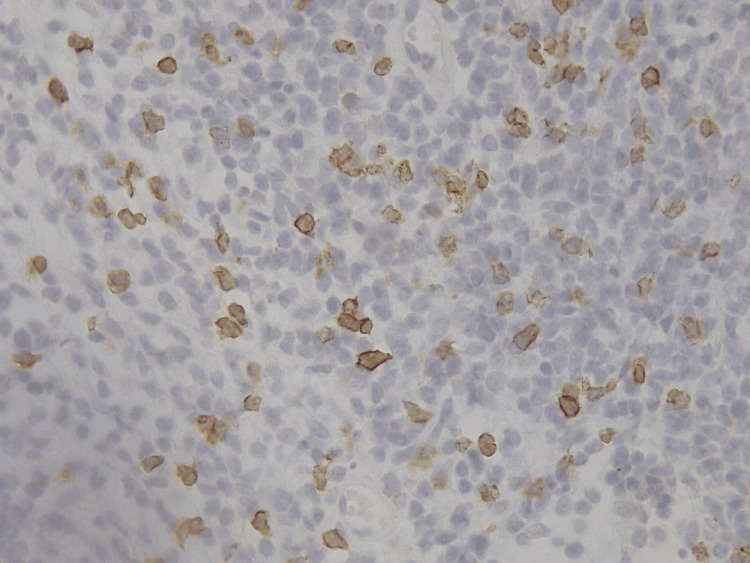
Immunohistochemistry – CD8 Immunohistochemistry for CD8 showing membranous staining in neoplastic lymphocytes (brown chromogen) within the dermis, with hematoxylin counterstain. Approximately 15% of the lymphoid population is positive, with an intensity of 75%. Co-expression of CD4 and CD8 in the same lesion is an uncommon finding in mycosis fungoides and may have prognostic implications. Skin biopsy from the ulcerated nodular lesion on the left thigh. Original magnification ×400.

He returned in 2025, now presenting a significantly worsened dermatosis on the same location (left thigh), described as a large, infected, ulcerated nodular lesion measuring approximately 12 × 18 cm, as shown in Figure 1. On examination, the lesion exhibited irregular raised borders, areas of black necrotic slough, yellow fibrinous exudate, and surrounding erythema, with associated foul odor and pain that limited mobility. Two biopsies performed at that time revealed epidermal and dermal lymphoid infiltrates with an immunophenotype positive for CD3, CD4, and CD8, negative for CD30, negative for CD7, and demonstrating clonal T-cell receptor rearrangement. Proliferative activity was assessed by Ki-67 immunostaining, which showed approximately 25% positive cells with 100% nuclear staining intensity, indicating a moderate proliferative index. This finding, although not specific, supports the presence of an active neoplastic process and is consistent with advanced-stage MF. Immunohistochemical staining for CD4+ and CD8+ is illustrated in Figure 2 and Figure 3, respectively.

Further extension studies revealed no evidence of lymph node or visceral involvement, and no Sézary cells were detected in the peripheral smear. Flow cytometry revealed a small clonal T-cell population consistent with B1 blood involvement, classifying the disease as stage IIIA (T3N0M0B1).

**Figure 4:**
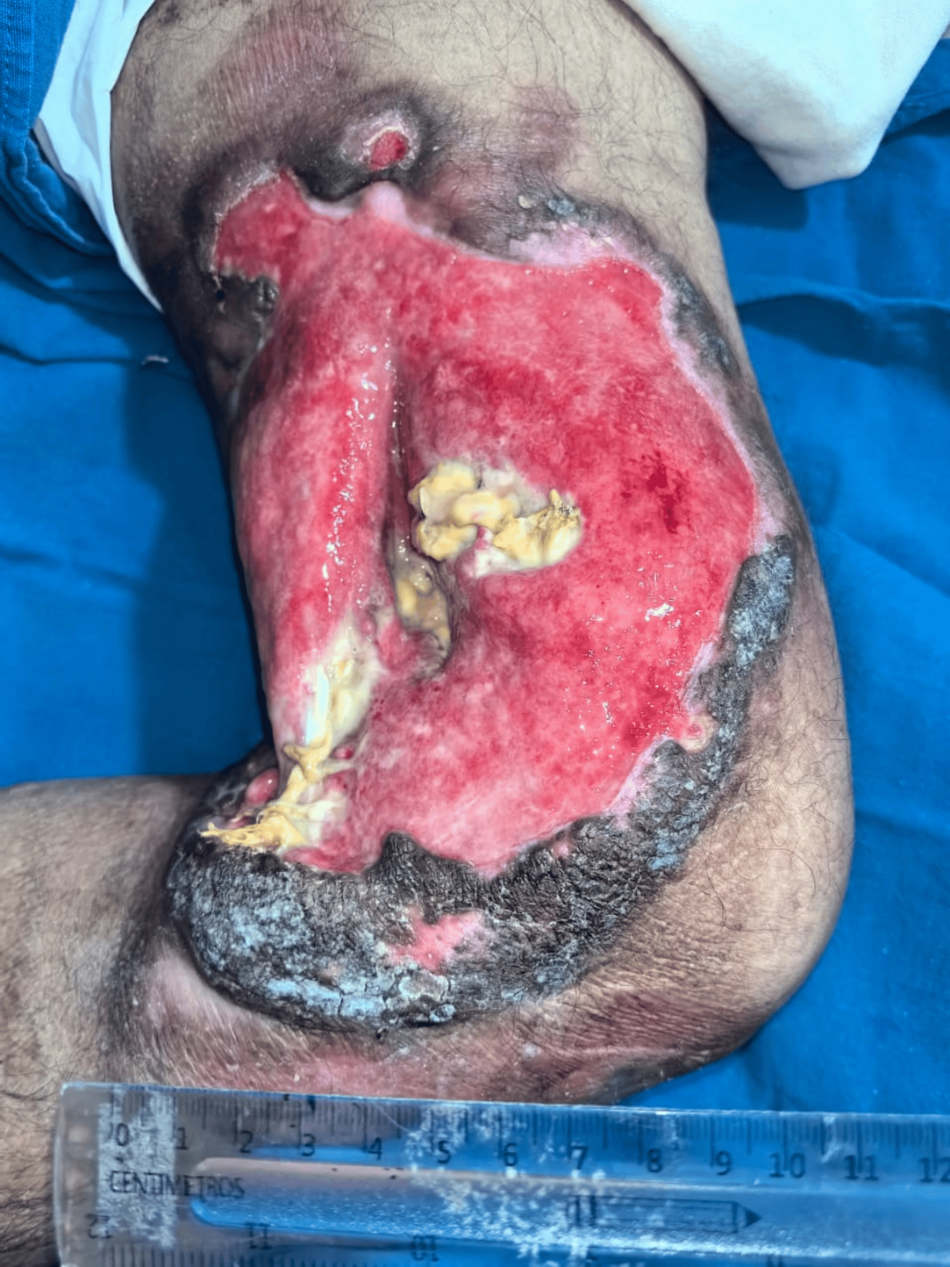
Same patient after therapy. Same lesion after two cycles of CHOP therapy, reduced to approximately 8 × 12 cm, showing a clean granulating base (red tissue), markedly decreased necrotic material, and less inflammatory exudate.

The patient initially received immunosuppressive therapy with methotrexate as first-line treatment with no response. After two cycles of the CHOP regimen ( cyclophosphamide, doxorubicin, etoposide, and vincristine) combined with gamma globulin, the lesion demonstrated marked clinical improvement. Necrotic tissue and thick fibrinous exudate were largely replaced by healthy granulation tissue, and the lesion dimensions decreased from approximately 12 × 18 cm to about 8 × 12 cm. Inflammation, malodor, and exudate were significantly reduced, and the patient reported substantial pain relief and improved mobility. These objective changes are reflected in the updated figure legends, which now provide a more detailed visual description for clarity (Figure 4).

## Discussion

MF is the most prevalent form of CTCL and is known for its indolent yet progressive course. Diagnosis can be delayed, particularly in the early patch and plaque stages, due to the nonspecific appearance of skin lesions. Disease progression leads to the development of tumor-stage lesions, which carry a poorer prognosis [3].

While MF generally presents with a CD4+/CD8−phenotype, some cases demonstrate co-expression of CD4 and CD8. This dual-positive immunophenotype is unusual and not well understood. Some literature suggests an association with slower disease progression and better clinical outcomes [3]. At the same time, other studies do not find significant differences in prognosis [5]. The characteristic epidermotropism observed in MF is thought to be mediated by adhesion molecules and skin-homing receptors expressed on neoplastic T [4]. The disease predominantly affects males, with a male-to-female ratio of approximately 1.6:1 [5]. The incidence of MF is approximately 5.6 cases per million people [6].

The presence of extracutaneous disease, including lymph node, blood, or visceral organ involvement, is strongly associated with worse survival outcomes [2]. In this specific case, the patient presented with T3 stage (≥1 tumor measuring ≥1 cm), N0 (no palpable peripheral lymph nodes), M0 (no visceral organ involvement), and B1b (clone-positive), which is a tumoral stage, one of the three typical clinical stages of MF. Tumors represent an advanced stage, appearing clinically as nodules measuring ≥1 cm in diameter. Histopathologically, this advanced stage features dense, diffuse sheets of neoplastic lymphocytes in the dermis [7].

The CD30 marker has been suggested as a potential prognostic indicator in transformed MF, although its prognostic value remains uncertain. A review by Travaglino et al. noted that CD30-positive cases of transformed MF had less than half the risk of death compared to CD30-negative cases [6]. Imaging tools such as PET-CT are often recommended from stage IIB onward, and targeted biopsies may be necessary to confirm systemic spread [8].

In establishing the diagnosis, other cutaneous lymphoproliferative disorders and inflammatory dermatoses were excluded. CD30-positive lymphoproliferative disorder was ruled out due to negative CD30 staining and the presence of strong epidermotropism, a hallmark of MF, rather than the cohesive dermal infiltrates typical of CD30+ disease. Chronic plaque psoriasis and nummular eczema were excluded histologically, as there was no psoriasiform epidermal hyperplasia or spongiosis, respectively [3,7,8].

Treatment decisions are based on disease extent, symptom burden, and patient comorbidities. For early MF, management often involves topical therapies and phototherapy. Advanced-stage disease may require systemic treatment, including methotrexate, retinoids, or multi-agent chemotherapy [8,9]. In this case, methotrexate was ineffective, and CHOP chemotherapy was chosen due to accessibility and prior evidence of efficacy in other lymphomas [1,10]. The patient demonstrated a clear and clinically significant improvement following treatment. CHOP inclusion in this regimen was determined by the institutional chemotherapy protocol for aggressive lymphomas and the limited availability of alternative agents for advanced MF in our setting. Published evidence supports the use of CHOP-based regimens in advanced cutaneous T-cell lymphomas [1,9].

For individuals in advanced stages (IIB to IV) without evidence of leukemia, bexarotene or methotrexate are frequently incorporated alongside prior treatment modalities. Patients with hematologic involvement are generally treated with either monotherapy or combination chemotherapy, utilizing agents such as CHOP, liposomal doxorubicin, gemcitabine, or similar multi-drug protocols [9].

Due to resource constraints and the socioeconomic situation of most Mexican patients, therapeutic choices often cannot strictly follow international MF guidelines [10].

## Conclusions

This case describes a rare presentation of MF with CD4/CD8 co-expression, managed successfully with CHOP in a resource-limited setting. This uncommon immunophenotype, reported in less than 5% of MF cases, coupled with the patient’s advanced stage and significant clinical improvement after therapy, underscores the importance of considering alternative regimens when standard treatments are inaccessible. From a diagnostic standpoint, the case reinforces the necessity of integrating detailed lesion morphology, immunohistochemistry with percentage, intensity, and distribution, and a focused differential diagnosis to distinguish MF from other cutaneous lymphoproliferative disorders such as CD30+ lymphoproliferative disease. Although the prognostic impact of this immunophenotype remains uncertain, it may be associated with a more favorable disease trajectory. Recognizing these uncommon variants is important for optimizing treatment strategies, particularly in contexts where access to standard therapies may be limited.

We acknowledge certain limitations, including the absence of long-term follow-up data and the lack of advanced molecular profiling beyond T-cell receptor clonality. Nevertheless, this case adds value to the literature by expanding clinical awareness of dual-positive MF, offering insight into real-world therapeutic adaptations, and highlighting the potential for meaningful tumor regression even in advanced disease under constrained conditions. Early recognition and precise immunophenotyping of atypical MF variants can enable timely, tailored treatment approaches, which are particularly important in healthcare settings with limited access to specialized therapies.

## References

[1] Khan N, Noor SJ, Horwitz S (2021). How we treat mycosis fungoides and Sézary syndrome. Clin Adv Hematol Oncol.

[2] Farabi B, Seminario-Vidal L, Jamgochian M (2022). Updated review on prognostic factors in mycosis fungoides and new skin lymphoma trials. J Cosmet Dermatol.

[3] Ding X, Chen J, Kuai L (2020). CD4/CD8 dual-positive mycosis fungoides: A case report and literature review. Medicine.

[4] Latzka J, Trautinger F (2023). Mycosis fungoides and Sézary syndrome - Review and outlook. J Dtsch Dermatol Ges.

[5] Korgavkar K, Xiong M, Weinstock M (2013). Changing incidence trends of cutaneous T-cell lymphoma. JAMA Dermatol.

[6] Travaglino A, Russo D, Varricchio S (2021). Prognostic significance of CD30 in transformed mycosis fungoides. Am J Clin Pathol.

[7] Alsayyah A (2020). Is it mycosis fungoides? A comprehensive guide to reaching the diagnosis and avoiding common pitfalls. Ann Diagn Pathol.

[8] Jonak C, Tittes J, Brunner PM, Guenova E (2021). Mycosis fungoides and Sézary syndrome. J Dtsch Dermatol Ges.

[9] Rofiq KS, Savitri M, Rahmatika A, Astari L, Ashariati A, Bintoro SU (2024). A case report: A successfully treated erythrodermic mycosis fungoides with CHOP chemotherapy regiment and narrow band-UVB. Curr Problems Cancer: Case Rep.

[10] National Academies of Sciences, Engineering Engineering, and Medicine; Health and Medicine Division; Board on Health Care Services; National Cancer Policy Forum (2017). Cancer Care in Low‑Resource Areas: Cancer Treatment, Palliative Care, and Survivorship Care: Proceedings of a Workshop. Cancer Treatment, Palliative Care, and Survivorship Care: Proceedings of a Workshop. Washington (DC): National Academies Press (US.

